# The inappropriate use of proton pump inhibitors and its associated factors among community-dwelling older adults

**DOI:** 10.1016/j.heliyon.2021.e07595

**Published:** 2021-07-15

**Authors:** Mohammad Rababa, Abeer Rababa'h

**Affiliations:** a Departement of Adult Health Nursing, Faculty of Nursing, Jordan University of Science and Technology, Irbid, 22110, Jordan; b Department of Clinical Pharmacy, Faculty of Pharmacy, Jordan University of Science and Technology, Irbid, 22110, Jordan

**Keywords:** Cognitive impairment, Comorbid burden, Cross-sectional, Older adults, Proton pump inhibitors

## Abstract

**Objectives:**

Little is known about the inappropriate use of proton pump inhibitors (PPIs) and how mild cognitive impairment (MCI) and high comorbid burden relate to the inappropriate prescribing of PPIs. Therefore, the current study aimed to examine these associations among community-dwelling older adults in Jordan.

**Method:**

This cross-sectional study was conducted on 215 community-dwelling older adults from three local healthcare centers located in Irbid, Jordan. Data about PPI use, including the name of medication, dose, frequency, duration, and indication, were collected retrospectively from a review of the participating older adults’ medication cards for November and December 2019. The collected data were evaluated based on the Food and Drug Administration (FDA) guidelines. MCI was measured using the Arabic version of the Montreal Cognitive Assessment, and comorbid burden was measured using the Cumulative Illness Rating Scale for Geriatrics.

**Results:**

Forty-seven percent of the participants were found to have taken a PPI, with 68 % having taken one for a longer period than recommended by the FDA. Older adults with MCI or high comorbid burden were found to be more susceptible than other older adults to the long-term use of PPIs. The logistic regression revealed that MCI is a statistically significant predictor of inappropriate PPI use (p < 0.001).

**Conclusion:**

Inappropriate PPI use is common among community-dwelling older adults in Jordan, with a significantly higher prevalence of inappropriate PPI use in people with MCI than in people with normal cognitive abilities. Future intervention studies are highly recommended to encourage optimal prescribing of PPIs for community-dwelling older adults.

## 1 Introduction

The inappropriate prescribing of medications is prevalent among older adults, a population with multiple comorbidities, cognitive impairments, and communication deficits [1]. This high prevalence is due to age-related physiological changes that affect homeostasis, pharmacokinetics, and pharmacodynamics in older adults [2]. The inappropriate prescribing of medications includes (a) the use of medications for longer than recommended, (b) drug-drug interactions and duplicate prescriptions, and (c) high risk-benefit ratios of drugs [3]. Many studies have reported the inappropriate prescribing of medications among community-dwelling older adults in both Western and Eastern countries [4, 5]. For example, medication prescriptions for older adults constitute 35–85% of the total number of medication prescriptions made in the Netherlands each year [4], and in Malaysia, the prevalence of polypharmacy among older adults is approximately 31.8% [5]. However, to the researchers’ knowledge, a very limited number of studies has examined the inappropriate prescribing of medications among community-dwelling older adults in Jordan [6, 7].

Proton pump inhibitors (PPIs) are among the most commonly prescribed drugs for gastrointestinal diseases (e.g., dyspepsia, gastric ulcer, and gastroesophageal reflux disease (GERD), which are highly prevalent among older adults [1]. Moreover, high body mass index and the high comorbid burden of chronic illnesses, which increases with age, are predictors of gastrointestinal diseases [8]. PPI use among community-dwelling older adults has multiplied over the last decades [9]. One of the most commonly used PPIs is omeprazole, taken by older adults worldwide [1]. A study by Voukelatou et al. found that 61.4% of the PPIs prescribed in Greece in 2019 had been inappropriately prescribed for older adults for non-evidence-based medical indications, with community-dwelling older adults being more susceptible than other age groups to the inappropriate prescribing of PPIs [2]. Another study revealed that approximately 60% of community-dwelling older adults were taking a PPI for non-evidence-based indications [10]. The inappropriate use of PPIs is associated with serious negative health outcomes, including pneumonia, osteoporosis, fractures, colon malignancy, and vitamin B-12 deficiency [11]. Moreover, it is estimated that around £2 billion are spent each year on the inappropriate prescribing of PPIs worldwide [12].

The long-term use of PPIs among people with cognitive impairment is a highly prevalent issue worldwide [10]. For example, in a study by Rababa et al. [1], around 92.5% of nursing home (NH) residents with dementia were found to have used at least one PPI for longer than recommended by the Food and Drug Administration (FDA). Evidence from the literature has revealed conflicting findings regarding the correlation between the risk of developing dementia and PPI use [13, 14, 15]. According to Hussain et al., there is no significant association between the long-term use of PPIs and the increased risk of developing dementia [14]. Meanwhile, a recent systematic review concluded that a significant correlation was found between being inappropriately prescribed a PPI and the risk of developing dementia [16]. However, the systematic review had some conflicting findings and methodological issues, which limit the reported association between PPI use and dementia [16]. Tai et al. found that the risk of developing dementia in older adults increases in patients who use a PPI for longer than recommended by the FDA [15]. On the contrary, according to a study in Germany, there is a negative association between PPI use and the susceptibility to developing dementia [17]. The current study focused on examining the possible association between older adults whether having mild cognitive impairment (MCI) or not and the risk of them being inappropriately prescribed PPIs. Thus, we hypothesized in the current study that older adults with MCI are more likely than other older adults to be inappropriately prescribed PPIs or to take PPIs for longer than recommended by the FDA.

To our knowledge, no study has examined the association between older adults having MCI and the risk of them being inappropriately prescribed PPIs. This association may be considered logical, as older adults with cognitive impairment suffer from greater levels of physical and psychological co-morbidities and may receive generally more potentially inappropriate medications (PIMs) than their cognitively intact counterparts [18]. In general, there is a significant positive correlation between PIM use and dementia in older adults [19]. Koyama et al. found PIM use to be significantly higher in older adults with dementia compared to those without dementia [19].

Physical and psychiatric comorbidities are associated with drug-drug interactions, which cause gastric upset diseases, including GERD [20]. Although the association between high comorbid burden and the use of PPIs is logical, this association has not been well-documented in the literature. Older adults are particularly susceptible to the impact of high comorbid burden [21], and many older adults experience significant age-related physiological changes and vital organ function limitations which may affect their homeostasis, drug pharmacokinetics, and pharmacodynamics profile. Medical and psychiatric morbidities make the interaction between the body and drugs more even complicated. According to Koyama et al., there is a significant positive correlation between PIM use and comorbid burden in community-dwelling older adults [19]. The purpose of the present study was to examine PPI use and its associated factors among community-dwelling older adults in Jordan.

## 2 Methods

### 2.1 Design, setting, and participants

A descriptive, cross-sectional design, which followed the Strengthening the Reporting of Observational Studies in Epidemiology (STROBE) guidelines, was used in the current study. Three local healthcare centers located in Irbid, northern Jordan, were chosen by convenience. These healthcare centers provide primary and emergency healthcare services, nutrition support services, vaccination, and prescription initiation, adaptation, and renewal for people of the local community. All older adults who regularly visited the healthcare centers during the month of January 2020 for routine checkups and prescription renewals were invited to participate in the study. Of the 235 older adults who were initially approached, 215 (91.5%) agreed to participate and completed the study. The sample size was calculated using G∗power software for one-way ANOVA based on the following parameters: effect size of 0.25, probability of error equals to 0.05, power of 0.9, and number of groups equals to 3. The sample size calculation yielded a minimum required sample size of 207, and eight participants were included to control any drop out of participation.

### 2.2 Ethical approval

This study was approved (approval # 749–2019) by the Institutional Review Board (IRB) of the university, and the administrative office at each of the healthcare centers. All personal data of the participants were de-identified and kept confidential. The researchers assured the participants that all collected data would be kept private, that participation in the study was voluntary, and that they could withdraw from the study at any time without consequences. Following the IRB approval and before collecting any study-related data, written informed consent was obtained from each participant. In the case of illiterate or sensory-impaired participants, the researchers read and explained the consent form, and in the case of cognitively impaired older adults who were unable to make their own decisions, a family member provided written consent, and the participant provided verbal assent. All participants who provided consent or assent had the opportunity to have their questions answered.

### 2.3 Measurement

#### 2.3.1 PPI use

Data about PPI use, including the name of medication, dose, frequency (PRN vs. scheduled), duration, and indication, were collected retrospectively from a review of the participating older adults' medication cards for November and December 2019. Each medication card contained a list of the medications prescribed for the patient and had been completed by the patient's primary care provider. The numbers of other prescribed oral daily medications potentially associated with PPI use, including vitamin and mineral supplements, analgesics, antibiotics, cardiovascular drugs, and respiratory drugs, were also collected. Data collection sheets developed by the researchers were used to abstract the data. The researchers trained two research assistants on how to complete the data collection sheets and monitored the data collection process throughout the whole study. The researchers asked the research assistants to confirm with the patients whether they were taking the medications, including PPIs, listed on their cards and whether they were taking any self-prescribed PPIs. The researcher calculated the interrater reliability of the data collected for every 10% of the sample to minimize measurement errors. The researchers then reviewed the PPI prescription of each participant to determine its compatibility with the FDA guidelines for dose, duration, and indication [22]. Interrater reliability assessment was conducted by comparing the ratings of the prescription reviews of the researchers.

#### 2.3.2 Mild cognitive impairment

Mild cognitive impairment (MCI) was measured once following the consent procedure and using the Arabic version [23] of the Montreal Cognitive Assessment (MoCA) [24]. The MoCA includes questions about cognitive skills, including visuospatial/executive, naming, memory, attention, abstraction, detailed recall, and language. The total score ranges from 0-30, with a score of <30 indicating MCI. The MoCA score was treated as a binominal variable in this study and categorized as: yes = MCI and no = normal cognitive abilities. Several studies have demonstrated acceptable reliability and validity scores for the MoCA [25, 26]. The Cronbach's alpha reliability score of the Arabic version of the MoCA used in the current study was 0.82, which is very close to the original article (Cronbach's α = 0.79).

#### 2.3.3 Comorbid burden

Comorbid burden was measured once following the consent procedure and using the Cumulative Illness Rating Scale for Geriatrics (CIRS-G) [27]. The CIRS-G is considered the gold standard for comorbidity measurement in older adults [28]. The CIRS-G objectively captures 15 of the most prevalent clinical conditions rated on a 0–4 disease severity scale [28]. The CIRS-G was designed to provide a comprehensive review of body systems to identify clinical conditions and rate their severity in older adults. In the original article, the CIRS-G had a high internal consistency reliability score [27], and in the current study, the Cronbach's alpha reliability of the CIRS-G was also high (Cronbach's α = 0.89).

#### 2.3.4 Other measures

The body mass index (BMI) of each participant was measured using the following formula (BMI = kg/m^2^), where kg represents the participant's weight in kilograms, and m^2^ represents the participant's height in meters squared. Participants with 25.0 ≤ BMI <30 were considered overweight, and those with 18.5 ≤ BMI <25 were considered at a normal or healthy weight. Meanwhile, participants with a score of 30 or more were considered obese. Demographic data related to age, gender, level of education, and marital status were self-reported by the participants.

### 2.4 Data collection

Following the IRB approval, the researchers met with the administrators of each clinic to discuss the study procedure, eligibility criteria, and time/date of the site visit. Posters explaining the study purpose and eligibility criteria were placed on the front desk of each clinic and at the entrance to each clinic's pharmacy in order to invite older adults to participate in the study. All older adults, whether they were taking PPIs or not, who visited the local clinics for regular checkups or prescription renewals were approached by the research assistant and asked if they would be interested in participating. The researchers met with each participant in a quiet private room and asked several questions to measure the participant's cognitive abilities and to determine whether the participant had dementia or not. Then, the researchers asked for the participants' medication cards in order to collect data about PPI use and the use of other associated drugs. Information about the participants' comorbid burden was collected by reviewing their medical records in the clinic.

### 2.5 Data analysis

Data analysis was conducted using the Statistical Package for the Social Sciences (SPSS version 25.0 for Windows), a statistical software program (Armonk, NY: IBM Corp). Descriptive results, including the sociodemographic characteristics of the participants, PPI use, having dementia, comorbid burden, BMI, and the number of daily oral medications taken, were presented using descriptive statistics. Means and standard deviations were used for the continuous variables, and percentages were used for the categorical variables. Data were normally distributed based on the results of the Shapiro-Wilk and Kolmogorov-Smirnov tests. Independent t-tests, chi-square, and one-way ANOVA were used to analyze the differences between the participant groups (i.e., PPI users vs. PPI non-users) in terms of their sociodemographic characteristics, BMI, and use of other drugs associated with PPIs. Logistic regression was used to examine the predictors of PPI use among community-dwelling older adults after controlling for their age and BMI. Based on the literature, increased BMI and advanced age are significant independent predictors of dyspepsia, GERD, and Barrett's esophagus, which are the main indications for PPI use [29, 30, 31, 32, 33]. The significance level was set at p < 0.05 in the present study.

## 3 Results

### 3.1 Characteristics of the participants

Fifty one percent of the participants were female, 80.0% were married, 35.8% have not finished their high school, and 42.3% had a normal BMI; further, the participants ranged in age from 55 to 103 years (mean = 87.7, SD = 7.8). Sixty-three percent (n = 136) of the participants had dementia, and the mean MoCA score was 19.5 (SD = 6.4). The mean CIRS-G score was 2.6 (SD = 0.3), indicating a high comorbid burden. A detailed description of the participants’ characteristics is summarized in Table 1.

**Table 1:** Descriptive statistics of the participants’ characteristics & PPI use.

Characteristics	Frequency	Percentage
**Gender (N = 215)**		
Male	104	48.1
Female	111	51.4

**Level of Education (N = 215)**		
Less than 12 years	77	35.8
Completed high school	54	25.1
College	41	19.1
Graduated	43	20.0

**Marital Status (N = 215)**		
Married	172	80.0
Single	43	20.0

**BMI (N = 215)**		
Overweight	78	36.3
Obese	46	21.4
Normal	91	42.3

**Having Dementia (N = 215)**		
No	79	36.7
Yes	136	63.3

**PPI Use (N = 215)**		
No	112	52.6
Yes	103	47.4

**PPI Dosage Regimen Problem (n = 103)**		
Too High	3	2.9
Too Long (>12 months)	70	68.0

**PPI Name (n = 103)**		
Omeprazole	69	67
Pantoprazole	27	26.3
Lansoprazole	5	4.9
Rabeprazole	2	1.9

**PPI Duration (n =103)**		
8–12 weeks	10	9.7
12–24 weeks	22	21.4
More than 24 weeks	71	68.9

**PPI Indication (n =103)**		
No Indication	20	19.4
Eosinophilic esophagitis	1	1
GERD	60	58.3
GU	9	8.7
GU Prophylaxis	7	6.8
Heart Burn	6	5.8

PPI: Proton Pump Inhibitors; BMI: Body Mass Index; GU: Gastric Ulcer; GERD: Gastroesophageal Reflux Disease.

### 3.2 Prevalence of PPI use

As seen in Table 1, 47.4% of the participants were taking PPIs. Of these participants, 58.3% were taking PPIs for GERD, while 19.4% were taking PPIs for non-evidence-based indications. Further, 6% of the participants were taking PPIs for gastric ulcer prophylaxis. Only 2.9% of the participants were taking a higher dose of PPI than recommended by the FDA. However, 68.0% had been taking a PPI for longer than (>12 months) recommended by the FDA guidelines, and 66% had been taking a PPI for longer than two years (Table 1).

### 3.3 Comparisons based on the participants’ characteristics

Although there were no statistically significant differences in PPI use among the participant groups based on gender, marital status, or level of education, statistically significant differences in PPI use were found between overweight, obese, and physically fit participants, *X*^*2*^ (2, N = 215) = 6.22, *p* = 0.045 (Table 2). The current study found PPI use to increase with BMI. Also, a statistically significant difference was found between older adults who have MCI and those with normal cognitive abilities, *X*^*2*^ (1, N = 215) = 4.94, *p* = 0.026. As with regards to the use of other drugs associated with PPIs, significant differences in the number of analgesics taken orally per day were found between participants taking PPIs and those not taking PPIs (*t*_213_ = 2.42, *p* = 0.016). Therefore, PPI use was found to increase with the number of analgesics taken by older adults.

**Table 2:** Comparisons of demographic characteristics and number of oral drugs by PPI use group.

	Not Taking PPI	Taking PPI	*P-value*
	*Mean ± SD*	*Mean ± SD*	
**Analgesics**	1.31 ± 1.15	0.96 ± 0.90	0. 016∗
**Antibiotics**	0.09 ± 0.32	0.32 ± 2.11	0.246
**Cardiovascular Drugs**	2.25 ± 1.87	2.50 ± 1.93	0. 337
**Respiratory Drugs**	0.25 ± 0.69	0.36 ± 0.72	0.242
**Vitamins**	0.96 ± 1.10	1.18 ± 1.14	0.150
	**N (%)**	**N (%)**	
**Gender**			
Female	53 (46.9%)	58 (56.9%)	0.144
Male	60 (53.1%)	44 (43.1%)	

**Marital Status**			
Married	91 (80.5%)	83 (81.4%)	0.084
Single	22 (53.1%)	19 (18.6%)	

**Level of education**			
Not completed high school	40 (35.4%)	36 (35.3%)	0.112
Completed high school	28 (24.7%)	25 (24.5%)	
College	22 (19.5%)	20 (19.6%)	
Graduated	23 (20.4%)	21 (20.6%)	

**MCI**			
Yes	63 (56.3%)	73 (70.9%)	0.026∗
No	49 (43.8%)	30 (29.1%)	

**BMI**			
Normal	54 (47.8%)	37 (36.3%)	0.045∗
Overweight	42 (37.2%)	36 (35.3%)	
Obese	17 (15.0%)	29 (28.4%)	

∗p < 0.05; BMI: Body mass index; PPI: Proton Pump Inhibitors.

### 3.4 Predictors of PPI use

Participants taking PPIs were more likely to have MCI than participants not taking PPIs. The overall regression model was statistically significant (chi-square = 73.54, *p* < 0.001; Table 3). Seventy-six percent of the cases were correctly coded. After controlling for age and BMI, MCI (*p* < 0.001) was found to be a uniquely significant predictor of PPI use in the model. Older adults with MCI were 15 times more likely to be taking PPIs than those without MCI.

**Table 3:** Logistic regression predicting PPI use.

Predictor	*β*	*SE*	*AOR*	*95% CI*
Age	0.034	0.021	1.035	[0.993, 1.078]
BMI	0.498∗	0.227	1.646	[1.056, 2.567]
Comorbid Burden	0.371	0.131	0.663	[0.675, 1.660]
MCI	2.979∗∗	0.427	14.675	[8.521, 45.428]

∗∗p < 0.001; ∗p < 0.05; PPI: Proton Pump Inhibitor; BMI: Body Mass Index; MCI: Mild Cognitive Impairment AOR: Adjusted Odd Ratio.

## 4 Discussion

The present cross-sectional study focused on identifying the prevalence of PPI use among community-dwelling older adults in Jordan. Of the participating older adults, 47.4% were found to be taking PPIs, the majority of whom had been prescribed a PPI for GERD. Alarmingly, the results of the current study revealed that the majority of the participants taking PPIs had been doing so for longer than recommended by the FDA guidelines. About 66% of the participants taking PPIs had been taking them for longer than two years. Fortunately, only a minor percentage of the participants showed nonindicated PPI use and reported taking a higher PPI dose than recommended by the FDA guidelines for older adults.

The analysis showed that the inappropriate use of PPIs was related to the concomitant use of other drugs. High rates of PPI use were noticed among patients taking analgesics, whilst no differences were noted based on gender, marital status, or level of education. This may be explained by the fact that analgesics are associated with gastric ulceration and thus PPIs are widely prescribed as gastroprotective agents [34, 35, 36, 37, 38]. However, monitoring for comorbid conditions and evaluating the patient's clinical status are more effective methods than long-term PPI use for managing the adverse effects of polypharmacy on the stomach and providing the patient with an optimal and cost-effective therapeutic plan [39].

Interestingly, the current study showed PPI use to be higher among patients with high BMI than patients with low BMI. Previous epidemiological studies have illustrated an association between patients with high BMI and the risk of GERD and have considered a high BMI as being an independent risk factor for erosive esophagitis [40, 41]. This comes consistent with our findings, which support the link between a high BMI and the use of PPIs mainly indicated for GERD and erosive esophagitis. Further, this may explain the statistically significant differences we identified in PPI use between overweight, obese, and physically fit participants.

The current study revealed that 63% of the participants had MCI. Additionally, the analyses showed that participants who had MCI were more likely to be taking PPIs than participants who did not. This finding is consistent with evidence which suggests that long-term PPI use may impact cognition [42]. MCI was a statistically significant predictor in our regression model, which is consistent with previous research studies [43, 44]. According to Eshetie et al., the prevalence of PIM use, particularly PPI use, was significantly higher in people with dementia (PWD) than in people without dementia [10]. The high prevalence of PIM use in PWD is attributed to many factors, including cognitive impairments, high comorbid burden and associated polypharmacy, age-related changes in pharmacokinetics and pharmacodynamics, and the lack of effective communication between patients and prescribers [10].

Similarly, in a recent study, Danish community-dwelling PWD were 26% more likely than people without dementia to be prescribed PIMs [45]. Our study finding is also supported by a study in Australia, which included 547 nursing home residents and which showed that PWD were more likely than people without dementia to be prescribed PIMs (P = .002) [9]. Moreover, Eshetie, Nguyen, Gillam, and Kalisch Ellett reported a significantly positive correlation between PIM use and diagnosis with dementia in older adults [46]. However, our findings do not correspond exactly to the findings of previous studies, as this study focused specifically on investigating the inappropriate use of PPIs among community-dwelling older adults. Nonetheless, our findings contribute to the existing literature, indicating that PWD are more likely than people without dementia to be taking PPIs, a type of PIMs.

Given the high prevalence of inappropriate PPI use among the community-dwelling older adults in our study, future intervention studies which address this issue are highly recommended. An interventional study which examines the effectiveness of using step-down PPI therapy or deprescribing could be optimal for reducing the risk of inappropriate PPI use among older adults. A recent study demonstrated a positive association between step-down PPI therapy and better gastrointestinal outcomes, including reduced gastric acid output and improved GERD symptoms [39]. Also, deprescribing, which refers to the process of intentionally reducing or stopping a medication, is an effective and safe intervention for counteracting the inappropriate prescribing of PPIs [1]. A recent study demonstrated a willingness among older adults, particularly if recommended by their physician, to reduce or stop PPIs, therefore indicating the potential success of deprescribing interventions [47]. Moreover, educational interventions designed to increase patients’ awareness of the side effects associated with the long-term use of PPIs are recommended for empowering shared drug therapy-related decision-making between physicians and patients.

To the best of our knowledge, this is the first study to examine PPI use and its associated factors among community-dwelling older adults in Jordan or any other Middle Eastern country. However, the current study has some limitations. First, data about PPI use and use of other drugs were collected from patients’ medication cards, which may have not included all physician notes or information about over-the-counter drugs. Thus, this missing information may have caused bias in the measured outcomes or differences between the study groups. Therefore, the finding regarding the prevalence of PPI wrong dosing needs to be interpreted with caution and replicated before being applied to practice. Second, the generalizability of the study findings may be limited by the relatively small sample size and single geographical area selected.

## 5 Conclusions

The current study revealed that inappropriate PPI use is common among community-dwelling older adults in Jordan, with a significantly higher prevalence of PPI use in older adults with MCI than in those without MCI. Future intervention studies are highly recommended in order to encourage the optimal prescribing of PPIs for community-dwelling older adults in Jordan.

## Declarations

### Author contribution Statement

Mohammad Rababa: Conceived and designed the experiments; Performed the experiments; Analyzed and interpreted the data; Wrote the paper.

Abeer Rababa'h: Analyzed and interpreted the data; Wrote the paper.

### Funding statement

This work was supported by 10.13039/501100004035Jordan University of Science and Technology (grant numbers 20200065).

### Data availability statement

Data will be made available on request.

### Declaration of interests statement

The authors declare no conflict of interest.

### Additional information

No additional information is available for this paper.
